# Apoplexy

**Published:** 1820-06-01

**Authors:** 


### Medico-Chirurgical Review,

AND

### JOURNAL OF MEDICAL SCIENCE.

( Analytical Series )

Nec tibi quid liceat sed quid fecisse decebit

Occurrat mentemque domat respectus honesti. CLAUD.

VOL. I.] JUNE 1, 1820. [No. 1.

### I.

1. *A Treatise on Nervous Diseases.* By JOHN COOKE, M.D. F.A.S. Fellow of the Royal College of Physicians, and late Physician to the London Hospital. Vol. I. *On Apoplexy, including Apoplexia Hydrocephalica, with an Introductory Account of the Opinions of Ancient and Modern Physiologists respecting the Nature and Uses of the Nervous System;* read at the College, as the Croonian Lectures of the Year 1819. Octavo, pp. 469. 1819.

2. *Researches on the Pathology of the Brain in Apoplexy.* By JOHN ABERCROMBIE, M.D. Fellow of the Royal College of Surgeons of Edinburgh. Octavo, pp. 73. 1818.

3. *Considerations et Observations sur l'Apoplexie.* Par M. BRICHETEAU. Journal Complimentaire, du Dictionaire des Sciences Medicales. Aout et Octobre, 1818.

4. *Recherches sur l'Apoplexie.* Par M. ROCHOUX. Octavo, Paris, 1814.

5. *Nouvelle Division des Apoplexies.* Par M. SERRES, l'un des Medecins de l'Hopital de la Pitie, Chef des Travaux Anatomiques des Hopitaux, &c.

[Annuaire Medico-Chirurgicale, Vol. I.]

HUMANITY is not liable to a more awful visitation than the sudden and invisible stroke which, annihilating sense and motion, prostrates every faculty of the soul in the dust! In an æra when all uncommon phenomena were attributed to the direct influence of a supernatural agency, persons, thus instantaneously affected with apoplexy, were considered and denominated ἐμβροντητοι, *attoniti, syderati,* or planet-struck. Primitive simplicity did not entirely secure the ancients from this severe dispensation; but assuredly the disease is now infinitely more prevalent than in primæval times. There cannot, therefore, be a more interesting subject of inquiry than the Pathology, Etiology, and Treatment of Apoplexy; and under this impression, we have constructed the following article on the eclectic principles and plan of the leading paper in our last number on Hydrocephalus. This species of composition will, we trust, prove an interesting feature in the Journal, and a useful trait in its analytical character.

I. *Symptomatology.* Of the various *definitions* of apoplexy laid down by authors, that of Dr. Cooke appears to us the most simple, and yet comprehensive.

"It is a disease (says this learned physician) in which the animal functions are suspended, while the vital and natural functions continue; respiration being generally laborious, and frequently attended with stertor."

Although the attack is often sudden, yet, where patients have paid attention to their own sensations, there have usually been some of the following premonitory symptoms, as pain in the head, ringing in the ears, vertigo, disposition to somnolency, numbness of the limbs, or sense of formication; dimness of sight; flashes of light before the eyes, with swelling and watering of those organs; flushing of the face; turgidity of the jugular veins; trembling and faultering of the voice; failure of the memory; deep breathing; a particular change in the countenance, which Dr. Gregory thinks is owing to a slight degree of paralysis affecting the muscles of the face, and though very observable, is indescribable. The same experienced physician observes, that apoplexy sometimes approaches with a violent pain in the head and bowels, accompanied by nausea.

After more or less of the foregoing premonitions, the attack is ushered in, according to Dr. Abercrombie, in one of the three following forms.

"I. In the first form, the patient falls down suddenly, deprived of sense and motion, and lies like a person in a deep sleep; his face generally flushed; his breathing stertorous; his pulse full, and not frequent, sometimes below the natural standard; in some cases convulsions occur. In this state of profound stupor, the patient may die after various intervals, from a few minutes to several days, or he may recover perfectly without any bad consequence of the attack remaining, or he may recover with paralysis of one side. This paralysis may disappear in a few days, or it may subside very gradually, or it may be permanent; other functions, as the speech, may be affected in the same manner; and sometimes recovery from the apoplectic state is accompanied by loss of sight.

"II. The second form of the disease begins with a sudden attack of violent pain in the head; the patient becomes pale, sick, and faint, generally vomits, and frequently, though not always, falls down in a state resembling syncope; the face very pale, the pulse very small. This is sometimes accompanied by slight convulsion. In other cases, he does not fall down, the sudden attack of pain being only accompanied by slight and transient loss of recollection. In both cases, he recovers in a few minutes; is quite sensible, and able to walk; continues to complain of intense head-ach; after a considerable time, perhaps some hours, becomes oppressed, forgetful, and incoherent, and thus gradually sinks into coma, from which he never recovers. In some cases, paralysis of one side occurs; but in others, and I think the greater proportion of this class, there is no paralysis.

"III. In the third form, the patient is suddenly deprived of the power of one side of the body, and of speech, without stupor; or, if the first attack is accompanied by a degree of stupor, this soon goes off; he appears sensible of his situation, and endeavours to express his feelings by signs. In the farther progress of this form of the disease, great variety occurs; in some cases, it passes gradually into apoplexy, perhaps after a few hours; in others, under the proper treatment, the patient recovers perfectly in a few days. In many cases, the recovery is gradual, and it is only at the end of several weeks or months that the complaint is removed. In another variety, the patient recovers so far as to be able to speak indistinctly, and to walk, dragging his leg by the most painful effort, and after this makes no further improvement. He may continue in this state for years, and die of some othor disease, or he may be cut off by a fresh attack. In a fifth variety, the patient neither recovers nor passes into apoplexy; he is confined to bed, speechless and paralytic, but in possession of his other faculties, and dies gradually exhausted, without apoplexy, several weeks or months after the attack." *555.*

Dr. Gregory, in his lectures, observes, that a fit of apoplexy perfectly resembles a state of extreme intoxication; from which, in fact, it cannot be distinguished, excepting by the actual cautery, which will rouse, in some degree, the inebriate, but has no effect on the apoplectic patient.

In the severer degrees of this disease, the function of respiration is generally much embarrassed, slow, and laborious at the beginning of the paroxysm; frequent, weak, and irregular towards the fatal termination. Stertor, though not always, is very commonly present; and some of our ablest physicians measure the violence and danger of the disease, by the degree of the stertor. This last remark is equally applicable to the frothy saliva, or foam, excreted from the mouth, and sometimes blown away from the lips with considerable force. In respect to the *pulse*, our experience tallies with the observation of Dr. Cooke, that it is "at first regular, strong, full, and slow, beating from 55 to 65 times in a minute; but, as the disease advances, it becomes weaker, and more frequent; and, in the end, irregular or intermitting." Dr. Gregory, in his Lectures,* observes, that "it is a fatal sign when the pulse is first small, and afterwards becomes very full." A cold clammy sweat is, he thinks, in general, a fatal sign. More frequently, however, the temperature of the surface is above par, and accompanied by copious perspiration. Fever is seldom an accompaniment of apoplexy, though the blood drawn often shews the inflammatory buff. Dr. Gregory has seen the disease carried off by the supervention of acute fever.

During the fit, the internal functions do not appear to be much disturbed; at least the secretions and excretions, which are nearly natural.

"When apoplexy terminates fatally, (says Dr. Cooke) as the disease proceeds, the abolition of sense and voluntary motion seems to become more complete; the respiration and pulse more weak and irregular; cold clammy sweats affect the face and whole body; the features shrink, and convulsions supervene, which terminate in death." *Vol. I. p.* 175.

The duration of a fit of apoplexy is various; but we agree with Dr. Cooke in thinking that many of those *sudden deaths* attributed to apoplexy, "depend upon some affection of the heart, or upon the rupture of some blood-vessel larger than those of the brain." Genuine apoplexy, Dr. Cooke believes, seldom destroys life in less than one or two hours.—The paroxysm usually lasts from eight to twelve, twenty-four, or forty-eight hours; sometimes for a still longer period. When not fatal, there is generally a greater or less degree of subsequent palsy; most frequently hemiplegia of the side opposite to that where the effusion is seated. This last, however, is not invariably the case; for the effusion is occasionally, though rarely, on the same side as the paralysis.

II. *Etiology.* This opens a wide and interesting field for

* A MS. copy of this distinguished teacher's lectures, in three quarto volumes, lately taken with great minuteness and accuracy, now lies before us.—Rev.

investigation; since on it depend the principles of Hygiene entirely. We shall follow the usual and natural division of the subject into predisposing and exciting causes.

*Predisposing.* The remark of Hippocrates, that apoplexies are *chiefly* generated between the fortieth and sixtieth year, has, it is thought, been confirmed by the experience of his successors. No age, however, is exempted from the disease, especially when induced by what may be termed mechanical causes. These are not connected with predisposition.

It may be necessary to observe, however, that Cullen and Portal consider apoplexies as much more frequent *after* sixty years of age than at any other period. Rochoux gives a table of sixty three cases, in the following order: between the age of 20 and 30, two cases—from 30 to 40, eight—from 40 to 50, seven—from 50 to 60, ten—from 60 to 70, twenty-three—from 70 to 80, twelve—from 80 to 90, one. This table then shews that in the twenty years from 40 to 60, the apoplectic period of Hippocrates, seventeen cases out of sixty-three occurred; whereas, in the next twenty years, viz. from 60 to 80, thirty-five cases out of sixty-three, or more than half of the whole number, and double that in the preceding twenty years, took place. This confirms the remark of Cullen and Portal.

The period of dentition in children is certainly a predisposing cause of the disease in them, which sometimes takes place. We cannot account for the altered balance of the circulation and excitability, which obtains in the middle period first noticed by Hippocrates; but such is the fact.—Cold and moisture, with sudden atmospherical vicissitudes, have been observed to predispose to apoplexy; and Baglivi asserts that it has sometimes prevailed epidemically in Italy, in consequence of unusual states of the weather. Plethoric constitutions and sedentary habits, short necks, indulgence in eating, drinking, and sleep, are well known predisposing causes; and the hereditary tendency to this disease is remarkable, through peculiarity of original organization. Intense thought appears to invite blood to the head, and keep up a local plethora there, which is favourable to apoplexy. Dr. Cullen considered obesity, as productive of difficult transmission of blood through the lungs, to be a predisposing cause of this formidable disease; Cheyne, "that the daily use of wine or spirits, even in what is considered a moderate quantity, will lead a man of a certain age and constitution to apoplexy, as certainly as habitual intoxication." And he concludes that "in nineteen cases out of twenty, the disease might be averted or postponed by temperance."

The suppression of accustomed evacuations, and the translations of gout are well known predisposing causes of apoplexy.

The apoplectic *form* has been remarked in all ages.

"A large head, (says Bricheteau,) a florid complexion, short and thick neck, broad shoulders, ample chest, globular abdomen, short stature, strong members, and general embon point, are the prominent features of the apoplectic figure." "If with these (says Bombier) are associated those habits which make the head a focus of excitement and vascular fluxion, at the same time that the other organs are left inactive, the sensorial functions acquire a remarkable predominance, in strength and activity; but too often to the ruin of the individual. This is frequently exemplified in the persons of those who give themselves up to immoderate study, uncombined with a sufficient quantum of sleep and corporeal exercise."

*Exciting Causes.* Among these we may place those chronic disorganizations which take place within the cranium, and which, by obstructing the circulation, compressing the cerebral mass, or otherwise injuring the vital powers of the brain, excite that state called apoplexy. Yet, perhaps, they might, with equal propriety, be considered as predisponent causes only; since, as they are permanently present, it is evident that some other exciting causes are necessary to produce the disease.

The principle exciting causes then are, violent passions, great exercise, insolation, intemperance in the warm bath, food and drink,—in short, whatever occasions a more copious current of blood to the head, or impedes the return of blood from it. And, in this place, may be noticed, what has been too much overlooked, the excitement of apoplexy by certain organic diseases of the heart, especially active enlargement of the left ventricle, for which we refer to our seventh number, for January last, page 343 to page 345. But every disease of the heart, whether of an active or passive nature, must produce derangement of the circulation, and either drive the blood with too great momentum towards the brain, or retard the venous return therefrom, thus proving an exciting cause of apoplexy in the predisposed. As cardiac affections have confessedly increased of late years, as well as apoplectic, it is reasonable to infer that the former have contributed to the production of the latter.

Extreme cold applied to the body generally has a tendency to induce an apoplectic sleep, from which the patient seldom wakes.

III. *Pathology.* A man falls down with all the symptoms of apoplexy, but by bleeding, purging, and other means, he perfectly recovers. Another dies, under apparently similar circumstances and treatment, and, on dissection, extravasation of blood is found within the head. A third dies of apoplexy, and only serum is effused. A fourth presents, after death, only a turgescence of the vascular system of the brain; while a fifth, who dies with every symptom of perfect apoplexy, presents, on dissection, no cognizable trace of lesion in the brain, or any other organ of the body.

These various and contradictory appearances, post mortem, would seem to offer an insuperable objection to any fixed pathology of apoplexy; or, at least, might apparently sanction a division of the disease into different species. Nevertheless, we will venture to maintain an identity of morbid state, or, in other words, of pathology, in apoplexy, whatever may be the appearances after death. We consider *pressure* on the cerebral mass, or its appendages, as the real *efficient* cause of the apoplectic phenomena, in every case.

In the first place, we may observe, that when authors state that apoplexy destroys life without leaving any cognizable mark of its existence after death, they should also state, that this is a comparatively rare occurrence. Not to multiply authorities on this point, we shall take the sepulchretum of Bonetus for an example. Of seventy-six cases of fatal apoplexy related in that work, *one only* failed to present effusion, congestion, or evident organic disease in the brain! Let us examine this single exception.

An old theologian, of sedentary habits, scorbutic disposition, and afflicted with dyspnœa, heaviness about the head, and great torpor, was suddenly stricken speechless and insensible, while on his knees in chapel. He was carried to bed, and Bonetus and other physicians summoned; but they found him "not only without sense, but without pulse and respiration, the whole body cold and rigid." Reperimus eum non modo absque sensu, pulsu et respiratione, sed toto corpore frigentem et plane rigidum." *Appendix, Observat.* lvii.

It is needless to say that there is not a single proof of apoplexy in the above case. The brain and its meninges were in a healthy state; but the lungs were discoloured and universally infarcted with a frothy ichor: "pulmones discolores et ichore spumoso per totum infarcti." *ib.* Indeed, in a note upon this case, Bonetus himself attributes the death rather to syncope than to apoplexy; and, for the following good reasons, which, by the bye, we submit to the perusal of Dr. Abercrombie, who appears to have drawn a *forced parallel* between syncope and apoplexy.

"Medici in arte exercitati confusuri non sunt syncopen cardiacam et apoplexiam, cum a mediocriter etiam in arte versato utriusque discrimen facile agnosci possit; nam in syncope extrema frigent, pulsus evanescit, atque respiratio penitus aufertur: in apoplexia vero abolitis actionibus animalibus nihilominus respirant, arteriæ pulsant, extrema calida utplurimum deprehenduntur."

Thus, then, in the whole sepulchretum of Bonetus, we have no instance of fatal apoplexy, without manifest cause in the brain; and when we bear in mind that many, like as in the case above mentioned, are reported to have died of apoplexy, whose deaths were really attributable to other affections, we may fairly presume that we are speaking within bounds, when we say that forty-nine cases in fifty, of those where the *symptoms* of apoplexy are present before death, would exhibit, if accurately examined, extravasation, congestion, or other material cause of compression on dissection.

Moreover, we shall presently shew that the most dangerous and speedily fatal pressure may be made on the brain and origins of the nerves, by turgescence of the blood-vessels: and there is nothing more certain, than that this turgescence may so far subside, in the interval between death and dissection, as to leave no trace of its previous existence. This, in fact, we consider to be the natural and true solution of the difficulty respecting the cause of apoplexy, in those cases where the scalpel cannot detect deviations from the healthy structure. This explanation also applies to the majority of those slight apoplectic attacks which often vanish so easily under evacuations, or even without depletion, where the balance of the circulation is restored by nature or art, before irreparable injury is done to the sensorial functions.

M. Portal, in his "*Resultats de l'Ouverture des Corps*," says that "there is a fulness more or less considerable, in the blood-vessels of the brain, cerebellum, medulla oblongata, and often of the spinal marrow, with or without an effusion of blood or serum."

"I mention first (says Cheyne) the remains of an excited state of the *minute arteries* of the brain and its membranes, this probably being the most important, as it is the most unvarying appearance; then the extravasation of blood, probably the consequence of the excited state of the vessels; the *turgescence of the venous system;* the enlargement of the ventricles, partial or general; and lastly, the serous effusion."

It is evident that Dr. Cheyne means *distension*, by excitement of the arteries, for there is no other *post mortem* mark of this state; and it may be remarked that he notes also the turgescence of the *venous system*, which is to be borne in mind, when we come to examine the validity of Dr. Abercrombie's Hypothesis.

M. Bricheteau, in his able Memoir on Apoplexy, remarks, that even fatal cases of this disease present not seldom, only

"a general turgescence of the vessels, which congestion exerts on the encephalic mass a general compression sufficient to annihilate the nervous influence and destroy life."

"On n'y remarque assez souvent qu' une turgescence génerale des vaisseaux, laquelle congestion exerce sur la masse encéphalique une compression générale qui anéantit l'influence nerveuse et fait cesser la vie." p. 296. "This opinion (he says) has never been admitted or discussed in any work which he has consulted." *ib.*

In the second volume of the Monthly Series of this Journal, for 1816, we translated, condensed, and commented on Morgagni's two Epistles on Apoplexy, and at page 259, we brought forward a case from Morgagni, illustrative of this very subject; namely, the case of Peter Fasolati, an engraver at Padua, who died of apoplexy, and who was examined by Morgagni himself. No extravasation appeared; but, says he, "the whole *vascular* system of the brain was distended with fluid blood, in such a manner as I had never before witnessed. Even some small vessels, which usually are scarcely perceptible, were extremely large and turgid."

In our commentary on this case, page 260, the following passage appears.

"That apoplexy is frequently produced by *turgescence* of the vessels alone, was believed in ancient times as well as in modern." "By this means (says Galen) apoplexies are brought on, to wit, by much blood rushing tumultuously into the principle of animation." *(apud salium de affect. part.)* "It is indeed reasonable to suppose that in the majority of apoplectic recoveries, *congestion* only had taken place in the vessels of the brain. But if congestion give rise to the more favourable cases, it appears capable of producing the most desperate and instantaneously fatal ones also. If a sudden and great determination (so called) of blood takes place towards the head, and the vessels are uot unloaded either by artificial means, or by the rupture of their coats, or effusion of their serous contents, it is quite evident that the whole substance of the cerebrum and cerebellum becomes compressed, and the functions of life destroyed." *Med. Chir. Journal, Vol. II. for* 1816, *p.* 260.

So far we have completely anticipated M. Bricheteau, who relates a number of authentic cases to support the abovementioned opinions, and concludes with a sentence most singularly coinciding with the passage which we have re-stated from our own Journal.

"Ces congestions cerébrales, accompagnées de la plupart des symptomes de l'apoplexie avec epanchement, me paraissent devoir etre très-frequentes, et je crois que les malades qui en guerissent sont beaucoup plus nombreux que ceux qui y succombent. Dans ces variétés d'apoplexie, le coma et l'abolition des facultés intellectuelles m'ont toujours paru exister à un plus haut degré que dans les cas d'epanchement sanguin." 298.

He very justly observes that, in all probability, the greater number of cases reported as presenting no morbid appearances after fatal apoplexy, were cases of this kind, where either the vascular system of the brain was superficially examined, or where the turgescence had disappeared prior to dissection.

Dr. Fouquier, of La Charité, has lately stated a remarkable case, which he denominates *nervous* apoplexy, but which we think illustrates the effects of pressure from general turgescence, in a very satisfactory manner. A stout and well made girl of nineteen, missed a menstrual evacuation, and three days afterwards felt a weakness in the lower extremities, followed by paralysis of both the upper and inferior limbs, with vertigo, tinnitus aurium, &c. but undiminished mental faculties. When brought to La Charité she evinced all the signs of vascular plethora; therefore blood-letting, glysters, abstinence, and other antiphlogistic measures were prescribed. The paralysis of all the limbs, however, continued, with partial distortion of the mouth. In three or four days the respiration became laborious, and the pulse accelerated. She died on the seventh day from the attack.

"On dissection, the exterior vessels of the brain, as well as those of the plexus choroides were turgid with blood; and blood presented itself in numerous points of all the cut surfaces of the brain, the substance of which was very firm. There was no effusion or extravasation."*

Dr. Fouquier calls this *nervous* apoplexy; but really we can see no reason why the paralysis and ultimately death should not have here arisen from the *vascular* pressure on the source of animation, although we allow that the disordered state of the *nervous* system was the first link in the morbid chain.

This leads us naturally to an examination of Dr. Abercrombie's doctrine, "that apoplexy does not depend upon *pressure* or determination, but simply upon *interrupted* circulation."† We perfectly agree with this intelligent physician, that the doctrine of *determination* of blood to any particular part from an impulse, *a tergo*, is quite untenable. We have repeatedly, in this Journal, stated our opinion that the heart can have no elective power to distribute an undue proportion of blood to any particular viscus. The determination of blood must depend upon the state of the vessels themselves where it accumulates. There is every reason to believe that

---

* *Annuaire Medico-Chirurgicale, Vol. i. p.* 376.
† Ed. Journal, p. 581.

the vessels of the body, like the heart itself, have a power of self-dilatation, as well as contraction, as is instanced daily in what may be termed the erectile tissues. Here the blood is rather attracted than impelled to the part; as, for instance, in strong friction on the skin. This may be considered, in general, as a healthy and natural power of the vessels. But we have also reason to conclude that the vessels of a part often lose their tone, and become morbidly dilated with the blood which rushes from all parts to that where least resistance is given, as is seen where parts are bruised, burned, or where vessels are cut or ruptured. There is yet another way in which the balance of the circulation may be disturbed. If cold be applied to any part of the system, so that its vessels collapse, more blood must necessarily flow through some other part of the body; or the course of the blood may be mechanically obstructed in one channel, thus occasioning more to flow through another. These are the only ways in which we conceive the improper term "determination of blood" can be explained. So far we agree with Dr. Abercrombie; but when this acute physician tells us, that "apoplexy does not depend on *pressure*, but simply upon interrupted circulation;" and in the same breath explains this interruption, by calling it—"such a derangement of the circulation in the head, that *more* blood *enters* by the arteries than *can be transmitted* by the veins;"* then we not only demur to his theory, but call upon him for an explanation of an evident contradiction both in terms and fact. How the brain, which fills every part of the cranium, can escape *pressure* when more blood *enters* by the arteries than can be *disgorged* by the veins, we are utterly at a loss to conceive: and we venture to predict, that Dr. Abercrombie himself, when he views the dilemma into which he has fallen, will not attempt to reconcile the two passages above mentioned.

In apoplexy, we have no proof whatever of an *interruption* to the flow of blood through the brain. The carotids are pulsating; the jugulars swell on the application of a ligature, and bleed plentifully. In short, we have nothing to prove *interruption* of the circulation, but "a case which is entirely hypothetical,"† even by Dr. Abecrombie's confession. Not so the proofs of an *increased quantum of blood* in the vessels of the brain. In the majority of dissections, the vessels are found burst, or the blood extravasated by a kind of exhalation—a pretty solid proof of previous unnatural distension,

---

* Edinburgh Journal, p. 572. † Ed. Journal, p. 573.

and consequently pressure. In other cases, a serous effusion is found, which modern pathology has shewn to result, in general, from plethora of the vessels. In a very few cases, *comparatively*, nothing particular is found on dissection; and in *these*, we have at least as good reason to suppose there *had been* morbid distension of the vessels, as morbid interruption of the circulation. We do not deny that a mechanical interruption to the return of blood from the head may cause apoplexy, as ligatures round the neck, congestions of the lungs, diseases of the heart, &c.; but we contend, that in *these* cases also, it is *pressure* on the brain that produces the phenomena of the disease.

Dr. Abercrombie appears to have fallen into a great error, in supposing that because the brain is an inelastic substance, and therefore *incompressible*, it must also be exempt from *pressure*. According to his own hypothesis, we would ask, how the brain could escape *pressure*, when the return of blood by the veins was interrupted? Granting that it was completely inelastic, and incapable of being diminished in size by any force, still it would not suffer the less *pressure* on that account, whenever "more blood entered by the arteries than could be transmitted by the veins." On the contrary, the very property of inelasticity would subject it to a greater degree of *pressure*, when circumstanced as above.

When slow aqueous effusions in the ventricles *gradually* enlarge these cavities to an immense size, and frequently reduce the hemispheres of the brain to a mere shell, of which there are so many cases on record, is there no *pressure* then experienced by the cerebral mass, inducing absorption of it? We think Dr. Abercrombie will hardly reply in the negative. It is the *sudden* effusion of a fluid, sanguineous or aqueous, in apoplexy, and consequently the *sudden* pressure, which makes all the difference between the phenomena of this disease, and of chronic hydrocephalus internus.*

We shall now proceed to show how the encephalon may experience a morbid degree of pressure, without any recourse to the hypothesis of interruption. Water is incompressible; but if a bladder, filled with that fluid, be squeezed between the hands, will it be said that the water suffers no *pressure*, because it cannot be diminished in volume by the pressing power?

We know that the vessels of the brain, and every other part of the body, are endowed with vital and elastic powers,

---

* See a remarkable case of this kind in vol. i. of last Series, page 162.

calculated to confine the blood in its proper channels, without any assistance from the parts through which the vessels traverse. But what is the consequence when, from any cause, these vital or tonic powers of the vessels in the brain are diminished? Why, they immediately swell; and consequently *press* upon the brain — or burst, and extravasate their contents—or suffer the serous portion of the blood to exude; in which last two cases, the pressure is more *local* than in the first. Now we firmly believe that it is this diminution of vital power in the vessels of the brain, however induced, that forms the first link in the chain of causation in apoplexy, (where the disease is not owing to mechanical violence) the immediate consequence or *effect* of which is *pressure* on the encephalon, with its concomitants, the phenomena of apoplexy. On this principle we can easily explain why all the symptoms of this formidable disease, often vanish suddenly by a restoration of the balance of the natural vital powers in the vessels of the brain, before these vessels have given way, or poured out their serous contents—or before their turgescence has destroyed the functions of the encephalon beyond recovery.

Let us now, by the light of pathological anatomy, inquire where and why the vessels of the brain most frequently give way, and extravasate their contents.

The sagacious Morgagni, in the 18th article of his third epistle, observes, with some surprise, that in almost all the cases of sanguineous extravasation which he met with in the dissection of apoplectic bodies, the fluid, or the cavities which had contained it, were generally found in the corpus striatum, or thalami nervorum opticorum, or near one or both of them. Struck with these observations, M. Rochoux, (who has lately investigated the subject of apoplexy with great success) compared the result of his experience with that of Morgagni, and found them to be nearly similar. Thus, of forty-one dissections, M. Rochoux found twenty-six with extravasations in the corpus striatum, and two in the thalami nervorum opticorum, while only thirteen, or not half the number, were found in the various other parts of the brain.

"En effet, (says M. Bricheteau) l'etude approfondie du système vasculaire de l'encephale fait voir que des artères assez nombreuses pénètrent directement dans ces parties, sans se subdiviser dans la pie-mère, comme le font les autres vaisseaux qui servent à la nutrition du cerveau; par conséquent elles se trouvent à nu au milieu de la substance cérébrale, dont le peu de consistance la rend peu propre a soutenir l'effort impulsive du sang."*

---

* Journal Complimentaire, Octobre 1818.

"In fact, an accurate study of the vascular system of the brain teaches us that a number of arteries penetrate directly into these parts of it, [the corpus striatum, thalami, and vicinity] without ramifying on the pia mater, as the other vessels do, which are nutrient of the encephalon; consequently, they are situated in the middle of the cerebral substance, the consistence of which is little capable of supporting them against the impulse of the blood."

In illustration of this passage, M. Bricheteau observes, that if injections are pushed with force through the carotids of young subjects, artificial extravasations, analogous to those in apoplectic bodies, will be constantly produced in the corpus striatum and thalami nervorum opticorum, as was noted by that excellent anatomist Dr. Lallemand, formerly of the Hotel Dieu.

The reader will easily see how these facts come in to the support of the doctrine which we maintain, relative to the distension of the vessels and pressure on the substance of the brain as the efficient causes of apoplexy.

Sanguineous effusion is exceedingly rare in the cerebellum. Morgagni reports only a single instance;* Rochoux thinks it hardly occurs once in fifty times. Heurtault relates a case, and Bricheteau saw a single instance at the Hotel Dieu. The man had died instantaneously; and on dissection, a considerable effusion of blood was found in both lobes of the cerebellum.†

Blood is rarely effused, *in the first instance*, into the ventricles. During ten years' observation in the different hospitals, M. Bricheteau only saw two cases of this kind. The fluid is generally extravasated in the neighbourhood of the ventricles, and bursts into them by a ragged opening. In the two cases above mentioned, there was no rupture of the parietes of the ventricles, nor of any particular vessel. The blood was effused by a kind of exhalation. This sanguineous exhalation takes place in other parts besides the ventricles. It is sometimes, though rarely seen, on the surface of the brain, and most probably comes from the vessels of the pia mater, or arachnoid covering. M. Rochoux relates an interesting case of this kind in a man 67 years of age, who had been sometime paralytic. He was carried to the hospital, and died with all the symptoms of apoplexy. On dissection, six ounces of blood were found extravasated on the surface of the right hemisphere of the brain, which, at this place, was considerably depressed. The corresponding portions of pia mater and tunica arachnoidea were gorged with

---

* Epist. ii. Art. 22. † Journ. Comp. Oct. 1818. p. 292.

blood, but no ruptured vessel could be detected by the minutest examination.

An interesting point in the pathology of apoplexy and paralysis; namely, the means by which Nature sometimes preserves life and restores health, after sanguineous extravasation, has been too much overlooked in this country, and is not even noticed by Dr. Cooke. To Drs. Rochoux and Riobé we are indebted for the best investigation of this subject. Dr. Rochoux, in his chapter entitled "*Appreciation des lesions organiques observées après la mort des apoplectiques,*" states that,

"The extravasation is generally in the *substance* of the brain, and rarely on the *surface* of this organ. In the *former* case, the blood is contained in cavernous pouches, which Wepfer and Morgagni compared to aneurismal sacs, and which frequently open by rents into the ventricles, or on the surface of the cerebrum. The parietes of these caverns are very soft, tinged strongly by the blood, about a line or two in thickness, unequal, anfractuous, and evidently lacerated on their internal surface, and presenting flaky filaments when agitated in water. They are surrounded by a layer of cerebral substance, about three lines in thickness, of a pale yellow colour, and the consistence of thick cream, scarcely miscible with water. This layer becomes gradually blended with, and lost in the surrounding healthy brain."

The above applies to *recent* apoplexy. The following are the appearances presented by those who have recovered from apoplexies, especially if followed by paralysis.

"After the absorption of the blood, (says M. Rochoux) the parietes of the caverns above described, approximate, and, in some measure, cicatrize, by the intervention of a cellular and vascular connexion, forming various areolæ, between which is found a reddish, ichorous fluid, more or less abundant, and sometimes glutinous. These parietes are much denser than the rest of the brain, about a line or two in thickness, and of a yellowish brown colour. I affirm that these caverns are constantly found after apoplexy, terminating in paralysis; and their number always corresponds with the number of attacks."

M. Rochoux entertains an opinion, that the morbid state of the brain, described in the first of the above extracts, precedes the apoplectic effusion; and, in fact, *is* the cause of it, rather than the consequence. We think it probable that this may sometimes be the case; but cannot go the length of Rochoux's hypothesis. On the contrary, we should be inclined to view the morbid condition of the encephalon immediately surrounding the extravasation, as *generally* resulting from the violence and dilaceration offered to that delicate structure.

Cotemporaneously with Rochoux, M. Riobé published the results of a similar investigation, carried on while an élève interne at La Charité. These are his conclusions.

"1mo. That apoplexy, with extravasation in the substance of the brain, is sometimes curable.

"2do. That occasionally there is a peculiar membrane developed around the sanguineous effusion.

"3tio. That this membrane secretes a serous fluid which bathes and dissolves the extravasated clot of blood.

"4to. That a great number of paralyses, caused by sanguineous effusion in the brain, gradually disappear, in proportion as the fluid is resorbed."

M. Riobé supports these conclusions by eight cases and dissections, to which a great many others are added by M. Bricheteau, all proving not only the resorption of the effused fluid, but a re-union of the lacerated surfaces afterwards, by a kind of cicatrization.

M. Serres, of La Pitié, in Paris, has, for some time past, been directing his attention to apoplexy, and has collected a great number of interesting facts, and made some curious experiments on animals, in elucidation of this important disease. Tired out with the fruitless attempts at distinguishing sanguineous from serous apoplexy, and constantly mortified by the uncertainty of the prognosis, when put to the test on dissection, he asked himself this question—*Do apoplexies differ from one another in their symptoms?*—Keeping this question in view, he closely observed the phenomena of the disease, at the bed-side, and was not long in perceiving that apoplexies assumed two very different forms—one, simple and uncomplicated with paralysis; the other, constantly accompanied by loss of motion on one or other side of the body. Was this chance? One hundred cases of the disease must give an answer. Of these, twenty-one were simple, and seventy-nine complicated with paralysis. Dissection of the former class disclosed the following appearances. Sixteen presented *serous* effusion, either in the ventricles or circumvolutions of the *cerebrum*—one with a sero-sanguineous extravasation in the left lateral ventricle—two with a similar effusion between the arachnoid and pia-matral tissues of both hemispheres—two without any effusion whatever. In all these cases the brain was sound, but the meninges were altered in the following manner.

1mo. In those instances where the affection had continued long, and the serous effusion was considerable, the pia mater was injected; its vessels much dilated; the tunica arachnoidea opake, and thickened.

2do. In the case where the ventricle alone was the seat of the sero-sanguineous effusion, the arachnoides, slightly opake in the rest of its expansion, was *red* in the interior of the ventricle, with numerous miliary granulations scattered over its surface.

3tio. In the two cases where the sero-sanguineous effusion was on the surface of the hemispheres, the arachnoid membrane was sensibly inflamed.

4to. In the two cases without effusion, the tunica arachnoides had a dry and somewhat thickened appearance, with membrani-form exudations.

This constant correspondence between the alteration of structure in the two inner tunics and the effusion, Dr. Serres looks upon as a very presumptive proof that they are linked together as cause and effect. A multitude of other cases subsequently examined has brought him to the conviction that, in this species of apoplexy, the effusion results from an irritation thrown upon the pia mater, or tunica arachnoidea, or both.

The anatomical characters of this class of apoplexies are very different, according to our author, from those attending the complicated forms. In the latter, the substance of the brain itself is altered. Excavations are found in its structure, filled with blood of various appearances, according to the time which had elapsed between the extravasation and death; while the portions of brain immediately surrounding these excavations are found in a state of redness, induration, yellowness, and irritation. From the foregoing and other considerations, our author has been led to the following conclusions.

"1mo. When an apoplectic attack presents no symptom of paralysis, we may presume that its seat is in the meninges, and that the substance of the brain is not dilacerated or altered.

"2ndo. When, on the contrary, paralysis becomes complicated with apoplexy, it is no longer the meninges, but the encephalon itself which is the principal seat of the irritation.

"3tio. Serous, sanguineous, sero-sanguineous, and purulent effusions, are owing to irritation in the meninges, or the encephalon, or to rupture of arteries or veins which may take place during the apoplexy; that is, subsequent to the irritation.

"4to. If I am not mistaken, we may very properly designate apoplexes then, in the following manner: Where there is *not* paralysis, I would call the disease 'MENINGEAL APOPLEXY;' where there *is* paralysis, 'CEREBRAL APOPLEXY.' On a future occasion I shall treat of cerebellic apoplexies in a subsequent memoir. I conceive that it is possible to say, during life, which of these apoplexies we have at any time to treat. If we have simple apoplexy, all the members will be excitable when a proper stimulus is applied, and the seat of lesion is in the meninges. Is there, on the contrary, hemiplegia, or any deviation from the natural posture of the mouth —then we have cerebral apoplexy.' 277.

M. Serres observes that, although the profound stupor, in which apoplectic patients are generally plunged, may render the excitability of the members somewhat equivocal; yet that the affection of the mouth is rarely, if ever, wanting in the cerebral apoplexy.

M. Serres proceeds to make many important remarks on these two species of apoplexy. He has observed the meningeal apoplexy to attack principally before the fifteenth, and after the sixtieth year, the female sex being more liable to it than the male. Out of forty-one cases of this species, thirty-three were females, and eight males. And the registers of the SALPETRIERE and BICETRE make the preponderance still greater on the side of the female sex.

The invasion of meningeal apoplexy is almost always slow, and preceded by various premonitory symptoms, of which, the most constant is a general torpor of the system, a disinclination to mental exertion, a fatigue from the least intellectual labour, obtuse perceptions, overwhelming sleep, respiration and circulation slower than in health; vital heat below the normal point; diminution of the secretions; derangement of the digestive functions.

If meningeal apoplexy, however, succeeds the suppression of an habitual drain from the system, or of a cutaneous eruption; if it comes on after a blow or fall on the head, the invasion is much more sudden; and, in these cases, there is a general pain in the head premonitory of the attack.*

In many apoplectic cases, under the care of our author, the disease stole on so imperceptibly, that it was thought the patients were in a tranquil sleep, when, in reality, they were completely apoplectic. How, says M. Serres, are we to distinguish apoplexy from sleep? In sleep, the respiration is slow and the circulation corresponding: in apoplexy, the equilibrium between these two functions is broken. The frequency of the pulse contrasts with the slowness of the respiration. The rhythm of the pulse may vary with the age or strength of the patient; but the discordance of action between it and respiration will always be found in apoplexy. The greater the degree of this discordance the greater the degree of the evil.

* Spontaneous vomitings sometimes precede the attack. *Vide Morgagni Epist. I. No.* 4.

In meningeal apoplexies, M. S. has remarked that the mouth is never drawn to one side; the patient lies in a right line in bed. If stupor be not present, the patient will present either hand, or move either leg, on being requested to do so. The nervous and muscular power, in fact, is equal on both sides of the body.

Of this class of apoplexies M. Serres makes five varieties: 1st. Meningeal apoplexy, without effusion; 2d. with serous effusion; 3d. with sero-sanguineous; 4th. with arterial rupture or dilatation; 5th. with rupture of veins.

1. In the *first* variety, (without effusion) M. Serres found, as was before hinted, the pia mater thickened and dry, the vessels somewhat distended, and the dura mater thickened in many places; the tunica arachnoides opake, and where it lines the ventricles, covered with whitish granulations, of an extraordinary form.

2. In the second variety of meningeal apoplexy, (serous effusion) the arteries and veins of the meninges were found distended, the whole of the pia mater covered with a lace-work of innumerable small vessels;* the arachnoid very opake, thickened, and covered in certain places with a whitish exudation particularly conspicuous along the principal venous trunks, over which it formed a kind of veil. The opacity and thickness of this tunic were much more considerable at the base of the encephalon, about the pineal gland, and in the ventricles, than elsewhere. In this variety, the plexus choroides is almost always altered from the natural texture, being distended, and presenting transparent cysts filled with a pellucid, sometimes a yellowish fluid, slightly saltish to the taste. At other times, this fluid was sanguineous, or sero-sanguineous; more rarely little clots of blood were found in their interior. The size of these cysts varies much. M. Serres has seen them as large as a small musket ball.

3. In the third variety (sanguineous effusion), the morbid alterations of structure in the pia mater were nearly analogous to those in the second variety; but here the arachnoid envelope was manifestly inflamed and red, without its vessels being very distinct. This appearance is peculiarly remarkable in the ventricles, where the principal foci of irritation seem to be situated.

4. In the meningeal apoplexies, with arterial rupture, all the arteries were found vastly distended. A twig or branch was found ruptured on one side, or broken quite across. More rarely there was a small aneurismal pouch, ruptured like

---

* Several very expressive plates of this morbid structure are given.

a common aneurism in other situations. M. Serres has seen the internal carotid aneurismal and burst, while yet enclosed in the cavernous sinus; and he lately presented to the Philomatic Society an instance of aneurism of the basilary artery.

5. The rupture of veins is a still more frequent occurrence than that of arteries. In all cases where coagulated blood is found in the ventricles, or between the meninges, without dilaceration of brain, we may rest assured that the effusion took place from a venous or arterial branch. The venous rupture frequently takes place in the plexus choroides, and the blood is inclosed in a thin cyst, or between the laminæ of the pia mater. Here M. Serres details a great many well authenticated cases, illustrative of the five varieties of meningeal apoplexy, above described, and which are extremely interesting and satisfactory, but need not here be introduced.

*Cerebral Apoplexies.* The attack of cerebral apoplexy is often sudden or instantaneous, particularly in men of full habit, short neck, corpulent structure, and who commit habitual excesses in wine or women. It is worthy of remark that, in general, a few minutes before the invasion, the brain exhibits an extraordinary excitation, accompanied by a facility of mental operations—often an energy of expression, far beyond the usual capacity of the individual. Sometimes a numbness of one side of the body or face, or a fixed pain in the head precedes the attack; but more frequently an embarrassed state of the tongue, or difficulty of pronouncing certain words or letters.

Apoplexy is not the invariable sequence of these premonitory phenomena. In this state of incubation (if we may be allowed the expression) the fit may often be warded off by regimen, exercise carried to fatigue, revulsives skilfully managed, or small sanguineous evacuations, especially from the hæmorrhoidal vessels; for instance, by leeches to the anus, or aloetic medicines if they produce an hæmorrhoidal discharge.

These evacuations are particularly necessary in those individuals where there is an habitual tendency of blood to the head, as evinced by troublesome pulsation of the carotids when in bed, sparklings before the eyes, in the dark, and unusual sounds in the ears. Woe be to them who neglect such warnings as these!

Whether these premonitions appear or not, the face, at the moment of attack, assumes an unusual hue; the cervical and facial veins swell, (particularly if the person be in a state of mental perturbation); the tongue falters; the sight is obscured; the hearing blunted; the patient loses feeling and consciousness, and falls down upon that side which is subsequently to become paralytic; an observation worthy of being attended to by the medical practitioner.

In a few hours after the invasion (if the brain have not already suffered laceration on some point of its various surfaces) the respiration becomes considerably slower than natural. The venous blood thus experiences a mechanical obstruction to its return to the heart, and the latter organ begins to react in proportion; the pulse accordingly becomes hard and frequent; the artery vibrates, as it were, under the finger; in short, the action of the heart is quickened in proportion as the respiratory process is retarded. This contrast between the functions of respiration and circulation, M. Serres observes, has not been attended to, though it is a phenomenon well worthy of the physician's consideration. The force and hardness of the pulse continue—the laceration of the brain takes place: then it becomes small, quick, and concentrated.

The respiration is equal on both sides of the chest during the first hours, sometimes days of the complaint; but ultimately the thorax becomes unequally dilated; one side of the chest is, as it were, struck motionless, while the other seems to redouble its activity. The thorax appears flatter on the inactive side than on the other. This symptom so generally precedes the hemiplegic phenomenon, that the latter may usually be prognosticated; and sometimes, M. Serres thinks, prevented by active measures.

The sensibility is sometimes equally obtuse in both sides of the body; but sometimes more markedly so in that side which is about to be paralysed.

At last, hemiplegia takes place. M. S. has passed whole days and nights at the bed-side of apoplectics, watching the precursory and concomitant phenomena connected with paralytic seizure. He has seen distortion of the mouth precede, several hours, certain convulsive movements of the side to be afterwards stricken powerless. Sometimes he has seen an almost tetanic rigidity of the whole side, previous to the hemiplegia. Sometimes the actual paralysis appeared first in the muscles of the mouth; sometimes in those of the extremities; but of the extremities themselves, the lower was always paralysed before the superior. It is a little remarkable, that *sensibility* sometimes continues in the paralytic limb; in general, however, the loss of sensation precedes and accompanies the loss of motion. M. Serres passes over the paralysis which occasionally affects the stomach and intestinal canal.

This subject he will take up in a future paper, when speaking of the treatment.

*Pathology.* M. Serres dissected, with the minutest care, 171 subjects who had died of cerebral apoplexy, with hemiplegia of upper and lower extremity at the same time. In every one of these the disorganization was seated in the *opposite* hemisphere of the brain. In La Pitié he also dissected 41 bodies, with the same result precisely. The records of Salpetriere and Bicetre confirm the above statement. When both sides are paralytic, the disorder is in both hemispheres. Finally, the paralysis sometimes extends to all parts of the body; the mouth is *not* drawn to either side, and the patient dies, as from asphyxia, or as animals who have the pneumogastric nerves of both sides divided. Dissection, in such cases, presents the extravasation in the substance of the tuber annulare, or burst from thence and spread along the base of the skull. M. Serres here brings forward a great number of important and accurately detailed cases, in elucidation of his positions, and to which we refer the reader who wishes to examine the proofs. Meantime we shall certainly look with considerable anxiety for the continuation of M. Serres' Memoire, on the morbid alterations of structure in cerebral apoplexy, and on the treatment of the disease.

IV. *Treatment.* The *doctrinal* views which we have taken of apoplexy, bear immediately on *practice*—indeed, we make a point of drawing our theories from facts, instead of first setting up the hypothesis, and bending facts to it afterwards. Let us, for a moment, see how Dr. Abercrombie's theory applies to the treatment. How is an *interrupted* circulation in the brain, resulting from a diminished calibre of the *veins,* at their exits from the head, to be remedied? By venesection, says our author. It appears to us very difficult to comprehend how a farther reduction of the *venous* mass of blood can restore the lost balance between the veins and arteries, when, upon Dr. A's hypothesis the balance was already on the side of the arteries! The plain state of the case is this; — first, we have no proof of interrupted circulation in the brain; secondly, we have no power, that we are acquainted with, to remove that interruption; and thirdly, we believe that did it really exist, in the way which Dr. Abercrombie describes, venesection would increase the evil, instead of lessening it.

Our author, indeed, appears somewhat aware of the inconsistency which might be here urged, and therefore " *thinks*

we make a more immediate impression on the carotid," by bleeding from the temporal artery. But this will not do. Every man of experience knows that the great object in apoplexy is to lessen, as quickly as possible, the whole volume of the circulation. Even Dr. A. in the face of his owu hypothesis, states, that "perhaps the first bleeding should be from the arm, from a large orifice, so as to make an impression upon the whole system."

But when we lay aside hypothesis, and allow facts to convince us that, whether in general turgescence of the vessels, or extravasations into the substance or cavities of the brain, there must be *pressure* on the encephalon, then we clearly see the quo as well as the *quomodo* of blood-letting, be it from the veins, the arteries, or the capillaries.

There may be *comparative* advantages in opening the temporal artery rather than the jugular or brachial veins; but we confess that we have not been able to appreciate them in actual practice. In all cases of *local* congestion, the nearer we take the blood from the part, the better; and this, we believe is the principal, if not the only superiority which temporal arteriotomy has over brachial venesection.

It is well known that Portal and Cullen, among others, preferred bleeding from the external jugular vein, and we believe it to be equally effective as from the temporal artery.

To produce the intended effect, as Dr. Abercrombie justly observes, the bleeding should be such as to powerfully affect the system, inducing weakness of the pulse and paleness. It must also be repeated at short intervals, as soon as these effects begin to subside. It ought ever to be remembered, that in dangerous diseases a variety of auxiliary means should be simultaneously employed with the principal measure, as *these*, though subordinate in their nature, are often decisive in their conjunctive effects.

Dr. Abercrombie appears to attach no importance to local, or capillary bleeding. We agree with him that bleeding by "a few leeches," is little better than a placebo ; but we cannot admit that cupping in the nape of the neck, or temples, is of no use. On the contrary, we think local detractions of this kind are indispensible auxiliaries.

The apoplectic patient should be placed in an airy situation, and cool air admitted. His posture should be that which least favours the gravitation of blood towards the head ; for the laws of hydraulics are not abolished in the living machine. All ligatures should be speedily relaxed ; and the legs and feet immersed in warm water, or rubbed with stimulating applications. Cold evaporating lotions should also be be applied to the shaven scalp,* and blisters between the shoulders.

Next to blood-letting, purgatives, by acting on a large secreting surface in the abdomen, are of great importance. Calomel and colocynth should be exhibited by the mouth; while sharp purgative glysters, as solution of aloes in warm water, ought to be repeatedly thrown up to invite the action of the cathartics on the intestines. Dr. Abercrombie relates several cases in which little effect seemed to be produced by bleeding; whereas an evident improvement took place after a free evacuation from the bowels. Our own experience is in unison with this observation.

Our endeavours are sometimes crowned with early success, and the apoplectic state is suddenly removed. In other cases, the coma does not begin to subside till after some hours, or even a day or two. Dr. A. truly remarks, that "in some cases, they (evacuations) may be used in the most active manner, so as to reduce the system as far as appears expedient or safe, without diminishing the coma; and after all, we may find, upon dissection, that the disease was still in the state of simple apoplexy;"—that is, without any blood having *burst* its boundaries, though the vessels, in such cases, are unquestionably put unnaturally on the stretch. The knowledge of this fact is to be borne in mind, since it induces us to persevere longer in our remedial measures than we otherwise would, in opposition to the dastardly maxim of Areteus —"cum ita se habuerint, honestam fugam capessere bonum est."

"It ought to be known (says Cheyne) that from six to eight pounds of blood have been taken from a person by no means robust, before the disease, which ended favourably, began to yield."

But although the distinction between sanguineous and serous apoplexy is an ignis fatuus, yet we agree with Dr. Abercrombie, in thinking that we are by no means authorized to treat all cases of apoplexy in the same manner.

"In the extent of our evacuations, a due regard is certainly to be had to the age and constitution of the patient, and to the strength of the pulse; but I think I have grounds for saying, that there are no symptoms which characterise a distinct class of apoplectic affections requiring any important distinction in the treatment; or, in

---

* Dr. Abercrombie recommends that cold water, in a full stream, should be directed upon the crown of the head, and received in a basin held under the chin, the patient being supported in a sitting posture. He gives an example of a girl quickly restored by this remedy, from a state of what he conceived perfect apoplexy.

other words, a class which, in their nature, do not admit of blood-letting."*

Dr. Cooke is also properly guarded in his expressions on this point.

"I confess, (says this learned and experienced physician) notwithstanding the positive opinions and directions of some modern physicians on this subject, I would not venture to persist in the abstraction of blood, if, after free and repeated bleedings, there was no apparent advantage; and, *à fortiori*, if symptoms of debility should supervene." *Vol. I. p.* 312.

Dr. Gregory, in his Lectures, adopts the division of serous and sanguineous apoplexy. In the *latter*, he remarks that "bleeding is almost the only chance the patient has for his life." "In the *serous* apoplexy, it does not do much good; but it never kills the patient." *M.S. Lect.*

Dr. G. directs the jugular vein to be opened in preference, and, in general, two veins to be opened at the same time, so that a large quantity of blood may be suddenly abstracted, and a strong impression quickly made on the vascular system.

"After general blood-letting, (says this veteran physician) topical bleeding is proper. I have seen very remarkable effects from it. Leeches are of no service, and are really trifling. The only plan is cupping; I have seen the cupping glasses rouse the patient, when general bleeding had produced no effect." *M.S.S.*

Many of our readers remember the acrid controversy, in the sixth and seventh volumes of the Medical and Physical Journal, respecting the propriety of administering emetics in apoplexy. Most of the arguments, on both sides, and among all writers, are founded on the theory of the disease, and action of vomiting embraced by the individual writer; and very few *facts* are brought forward by any party. Dr. Abercrombie remarks, that if the remedy is ever to be employed in any apoplectic disease, it is in "the state in which the system has been reduced as far as appears safe and expedient, by large and repeated evacuations, and yet the coma has not been removed. In this case, the operation of a mild emetic would probably be free from danger." 29.

Dr. Cooke conceives that,

"In the strong apoplexy, there may be danger of determining too much blood to the head by the act of vomiting; I therefore would not venture to prescribe an emetic, till the safer remedies had been

---

* Dr. Abercrombie speaks highly of strong frictions applied to the body generally.

unsuccessfully employed. But if, after free and repeated evacuations of blood, both general and topical, and the administration of glysters and other revellents, no signs of amendment should be perceptible, I would endeavour to excite the action of the vis medicatrix naturæ, by the exhibition of an emetic of speedy operation, such as the white or blue vitriols." 331.

Dr. C. witnessed one case of recovery from the disease, in a somewhat milder form, by this remedy, when bleeding, &c. had been prescribed without any good effect. When, soon after eating, the strong apoplexy has supervened, and spontaneous vomiting comes on, as not unfrequently happens, Dr. C. would have more than usual hope of success from this practice. He candidly confesses, however, that in two cases of this kind, he lately prescribed an emetic without any apparent advantage.

As the sentiments of Dr. Gregory, on this subject, may be interesting to most of our readers, we shall here again quote from his M.S. Lectures.

"In certain cases (says Dr. G.) vomits are proper; but it is difficult to ascertain the period of the complaint when they may be beneficial. They should never be given till after large evacuations by blood-letting. They are most proper where the disease proceeds from a surfeit; but even then, after bleeding. In *serous* habits, vomits are very efficacious; but they must be given in a double or triple dose, on account of the great insensibility of the system. I don't imagine there is that great danger from vomits which has been represented." *M.S. Lect.*

The action of nausea and that of vomiting are very different. The former lessens the power of the heart, and the latter determines the circulation to the surface. On this account they may both be useful in congestions of blood in any internal organ.

The use of stimulants in an early period of the disease must be decidedly injurious.

"But, perhaps, (says Dr. Abercrombie) we may make a distinction between the action of stimulants in a vigorous and plethoric state of the system, and their action when the system has been reduced by large and repeated evacuations. In sudden sinking of the vital powers, which we sometimes meet with in inflammatory disorders, particularly of the bowels, I have frequently given large quantities of wine, with the happiest effect, almost immediately after a violent inflammation has been subdued, and I never saw the inflammation renewed by it. I imagine there are conditions of apoplexy in which stimulants might be given with safety and advantage, but the practice requires much caution."

Dr. Cooke gives nearly the same evidence in respect to sanguineous apoplexy; but although he does not contend for a strict pathological distinction between this and the *serous* form, so called, yet he thinks, that when apoplexy occurs in old age, in leuco-phlegmatic temperaments, debilitated habits, and is attended with a pale countenance, a feeble pulse, and comes on gradually, the means used in the sanguineous form should be employed, *but with caution,* "when the symptoms warning us of the approach of the disease appear." In the fit itself, he recommends the pediluvium, frictions, sinapisms, blisters, cathartics, and other stimulating external applications, rather than blood-letting.

"In those cases (however) in which there appears to be a determination of blood to the head, and increased arterial action, blood-letting may, perhaps, be properly prescribed, although we may believe that the symptoms depend on an effusion of serum." *Vol. i. p.* 337.*

*Prophylactic Treatment.* Seeing that apoplexy is so very fatal, under any method of cure, it is incumbent on the medical practitioner to take every means of putting his patients, predisposed to this disease, on a preventive plan that may ward off such a dire calamity. It is by diet and exercise that we can most effectually prevent apoplexy. Repletion, from full meals of animal food, is a very frequent cause of the disease; and consequently, temperance is the best prophylactic. By regular exercise, carried to a sufficient extent, much advantage will be gained, But where these two prophylactics cannot be enforced, recourse must be had to such medicines as keep up an action on the bowels, particularly aloetics and the blue pill. Local or general blood-letting must also be occasionally applied; and where a drain can be established by means of an issue or seton, good effects may be expected to result. As we have shewn that affections of the heart very often enter into the etiology of apoplexy, we should attentively watch the state of the vascular system, and endeavour

---

* Although in these synthetical, and partly original articles, we attempt no regular analysis of the works which we place at the head of our paper, and consequently have little opportunity of descanting on their individual merits; yet we cannot avoid stating it as our opinion, that Dr. Cooke's publication, though professedly a compilation, is a work that is calculated to prove extremely useful to various orders of the profession, especially to students, and those who have neither time nor ability to search the wide field of medical literature, and glean the grain from the chaff. The talents and learning of Dr. Cooke are well known; and we have only to regret that this erudite and experienced physician has not interwoven still more of his own sentiments among the opinions of others. Rev.

to tranquillize the mind: in short, the prevention of apoplexy must almost entirely depend on avoiding its causes—and he who carefully studies *these* will be best able to lay down a proper system of Hygiene.

